# Alternative splicing of modulatory immune receptors in T lymphocytes: a newly identified and targetable mechanism for anticancer immunotherapy

**DOI:** 10.3389/fimmu.2024.1490035

**Published:** 2025-01-07

**Authors:** Shay Tzaban, Ori Stern, Elad Zisman, Galit Eisenberg, Shiri Klein, Shoshana Frankenburg, Michal Lotem

**Affiliations:** ^1^ The Lautenberg Center for Immunology and Cancer Research, The Faculty of Medicine, Hebrew University of Jerusalem, Jerusalem, Israel; ^2^ Center for Melanoma and Cancer Immunotherapy, Sharett Institute of Oncology, Jerusalem, Israel; ^3^ Hadassah Cancer Research Institute, Hadassah Hebrew University Medical Center, Jerusalem, Israel

**Keywords:** alternative splicing, cancer, immunotherapy, T lymphocytes, immune receptors

## Abstract

Alternative splicing (AS) is a mechanism that generates translational diversity within a genome. Equally important is the dynamic adaptability of the splicing machinery, which can give preference to one isoform over others encoded by a single gene. These isoform preferences change in response to the cell’s state and function. Particularly significant is the impact of physiological alternative splicing in T lymphocytes, where specific isoforms can enhance or reduce the cells’ reactivity to stimuli. This process makes splicing isoforms defining features of cell states, exemplified by CD45 splice isoforms, which characterize the transition from naïve to memory states. Two developments have accelerated the use of AS dynamics for therapeutic interventions: advancements in long-read RNA sequencing and progress in nucleic acid chemical modifications. Improved oligonucleotide stability has enabled their use in directing splicing to specific sites or modifying sequences to enhance or silence particular splicing events. This review highlights immune regulatory splicing patterns with potential significance for enhancing anticancer immunotherapy.

## Introduction

Biologists have long been puzzling how the human genome, which bears considerable similarity to lower eukaryotes, is responsible for the complex, sophisticated organisms it creates. Following Sharp and Roberts’ description of RNA splicing, Gilbert, in 1978, hypothesized that alternative splicing (AS) might be the missing layer that leads to the immense protein diversity despite the only 23000-gene human genome.

RNA splicing is a “cut and paste” process, removing introns and rejoining exons from the primary gene transcript, the pre-mRNA. The process relies on the biochemical uniqueness of RNA, which DNA lacks, of extensive flexibility and intrinsic catalytic activity. Small nuclear RNAs that assemble sequentially are directed to conserved sequences in the 5’(GT) and 3’(AG) splice sites on the primary transcript in an orderly manner. Together, the small RNAs and numerous proteins form the spliceosome. An adenosine in the intronic segment performs a nucleophilic attack on the 5′ end, cleaving the 5′ nucleotide (generally the “G” in a GT); a loop is then formed and removed. Following, the exon upstream of the removed intron is ligated to the 5′ end catalyzed by the spliceosomal RNAs and the ribonuclear proteins (RNPs).

**Figure 1:**
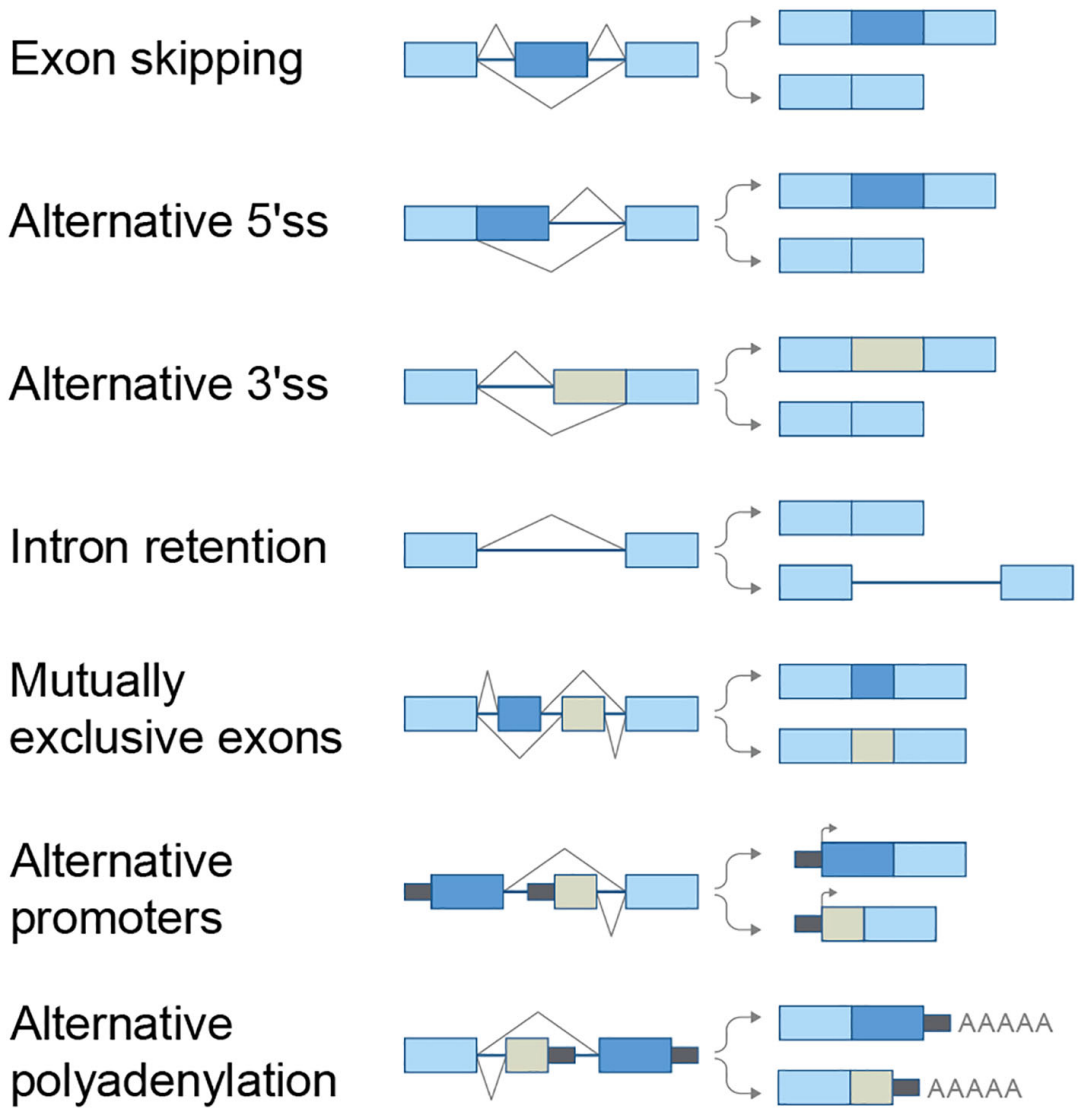
Alternative splicing patterns.

Alternative splicing produces variants that differ from the constitutive RNA transcript. It occurs parallel to the transcription process and produces several isoforms from one gene. Each isoform may lack an exon or part of an exon from either side of the constitutive exon. This pattern, called ‘cassette-type alternative exon’ or ‘exon skipping’, is the most common. Intron retention, uncommon in humans, occurs mainly in untranslated regions. See **Figure 1** for common splicing patterns.

AS involves 95% of the genes (1). With deep RNA sequencing becoming a more common read-out in experimental systems and longer RNA reads being produced, it is now clear that the pattern of RNA splicing is dynamically regulated and constantly changes (2).

The ratio between a constitutive transcript and its alternatively spliced isoforms depends on splice site recognition, its occupancy by spliceosomal RNPs, and regulatory RNA binding proteins (RBPs) (3). These RBPs bind or complement *cis*-sequences on the premature transcript on the intron or the exon. Sequences that promote spliceosome assembly at a splice site are called enhancers, and their RBPs are usually serine arginine-rich proteins. Sequences that reduce splice site recognition (silencers) attract heterologous nuclear RNPs (hnRNPs) (4). It is thought that enhancers usually act to generate constitutive mRNA, while the silencers yield AS isoforms. However, how the basic mechanism of AS varies from cell to cell and what determines it remains to be further elucidated.

Here, we focus on the role of AS in the T-cell immune response, particularly on anticancer immunity. This review aims to draw attention to new therapeutic opportunities in the functional distinctions between a constitutive protein/receptor and its splice isoforms. The motivation to unveil the intriguing mechanisms by which AS amplifies and regulates immune functions relies on reports from our group and others that RNA transcripts of the same immune gene can act in different directions or magnitudes in the immune context (5, 6). Thus, AS is an essential layer of immune regulation and a potential therapeutic target.

## Alternative splicing is a mechanism of dynamic adaptability

### Splicing event regulation

Although the prime outcome of AS is the fold increase in functionally distinct proteins compared to the number of genes, AS also plays a significant role in the most fundamental biological processes: evolution, differentiation, and adaptation. AS is a source of evolutionary development, a determinant of organ, tissue, and cell characteristics, and part of cellular adaptation to a changing environment (2).

The concerted manner by which protein production is shifted from one isoform to another yields the regulatory characteristics of AS. Its preferences differ among tissues and developmental states and respond to extracellular signals in a dynamic manner that precedes or synchronizes with gene transcription. In parallel to the dependency of intracellular processes on transcriptional activation, cellular events emerge from the shift in protein isoform ratios. How splicing events are concerted and what network cascades occur is a field of active research emphasizing health disorders and malignancies (7). The regulation of splicing events depends on both cis-acting regulatory sequences, located in introns or exons, and trans-acting splicing factor proteins that can strengthen or weaken the spliceosome’s recognition of the splice sites (8). These regulatory proteins belong to families of RNA-binding proteins, such as arginine–serine-rich (SR), heterogeneous nuclear ribonucleoprotein (hnRNP), and RNA-binding motif (RBM) proteins (9). They recognize specific regulatory sequences and enhance or inhibit the recognition of neighboring splice sites by the core splicing machinery (7). The expression level of the regulatory proteins is tissue- and state-specific (10), and they are subjected to regulatory splicing themselves (11).

From the evolutionary point of view, alternative splicing varies significantly among species. The insertion of multiple introns that separate exons has derived from ancestral genes and predated AS in eukaryote development. The option to skip exons was enabled by DNA mutations that may have resulted in splice sites with weaker binding affinity for spliceosomal components such as U1 small nuclear ribonucleoprotein (snRNP) (12). While the emergence of alternative splice sites contributed to protein diversity and is partly unique to a species, particularly when changing the reading frame, comparative genomics indicates that sequences that regulate RNA binding proteins are conserved and shared (13, 14).

Over 200,000 identified isoforms are reported in genome databases, and the majority of them lack functional annotation. Some well-studied examples show how events of retention or exclusion of specific domains may change protein cell localization, membrane anchorage, shedding of ectodomains, mRNA stability, and translational efficiency. The molecular alterations that emerge from AS may occur without any change in the level of the general gene’s transcript or before a change (15). Furthermore, the translational changes in reading frames may produce diverse translation outputs (13) and even insert poison exons, resulting in nonsense-mediated mRNA decay (NMD) and diminished protein levels (16). We can conclude that alternative splicing is timed and regulated in a manner that is not necessarily dependent on active simultaneous gene transcription.

## Alternative splicing in T lymphocytes

T-cell states are associated with preferential expression of specific splicing isoforms. A unique characteristic of T lymphocytes is that they transform within minutes from a stationary naïve or inactive state to intense activity. In their effector state, T cells must adapt to synthesize large amounts of cytokines, migrate, proliferate, lyse target cells, and address accelerated metabolic needs. Already in 2006, it was found that memory T-cells respond to antigenic stimuli faster than naïve cells by omitting exons 4, 5, and 6 from the extracellular part of the membrane phosphatase CD45. CD45 is expressed in T and B cells and, in its constitutive, full-exon inclusive state, is referred to as CD45RA. The CD45RO variant shows variable exclusion of exons 4, 5, and 6. CD45 dephosphorylates both inhibitory and costimulatory tyrosines of the Src-family kinases (17). Oberdoerffer et al. showed that the transition from the RA to the RO form depends on the activity of the splicing factor hnRNPLL (heterogeneous ribonucleoprotein L-like) (18). HnRNAPLL was suggested to be a master regulator in activated T cells, affecting not only CD45.

Before and in parallel to CD45, specific gene isoforms impacting T-cell function were discovered. Interesting events recorded in activated T-cells included the short isoform of CD28, which induces faster activation (19), splice variants of CD44 and CTLA4, which correlated with a higher risk of autoimmune disease (20, 21), and MALT1A, a paraprotease that integrates TCR activation with the downstream IKK/NF-κB pathway. Reminiscent of CD45, naïve T-cells express MALT1B, a splice isoform missing exon 7, while activated T-cells express MALT1A, which includes exon 7 and is associated with rapid NF-κB signaling and improved lymphocyte function (22, 23).

A landscape view of AS in immune cells was offered by Lynch et al. in 2004, preceding a complete landscape of the comprehensive gene involvement in this phenomenon (22). Although the list of spliced genes described to regulate lymphocyte activation was restricted, the diverse array of functions governed by splicing suggested the substantial ubiquity of this process (23).

In the last few years, analyses have focused on gene families and activation cascades as it becomes clear that AS affects most genes. An example is the production of anti-apoptotic splice isoforms of members of the BCL2 gene family in activated T-cells. Adding costimulation via CD28 increased the ratio of anti-apoptotic splice variants and augmented T-cell proliferation. Interestingly, the genes that displayed significant changes in their splice isoform ratios did not have the highest expression levels (24).

The concept of AS-induced effector transition is not limited to activated T lymphocytes but also plays a critical role in B cell affinity maturation. In these processes, poly-pyrimidine tract binding proteins PTBP1 and PTB3 are splicing factors that drive the appropriate expression of gene sets required to adapt B lymphocytes to antibody-producing cells (25).

### Splicing events that generate soluble isoforms of immune receptors

**Figure 2:**
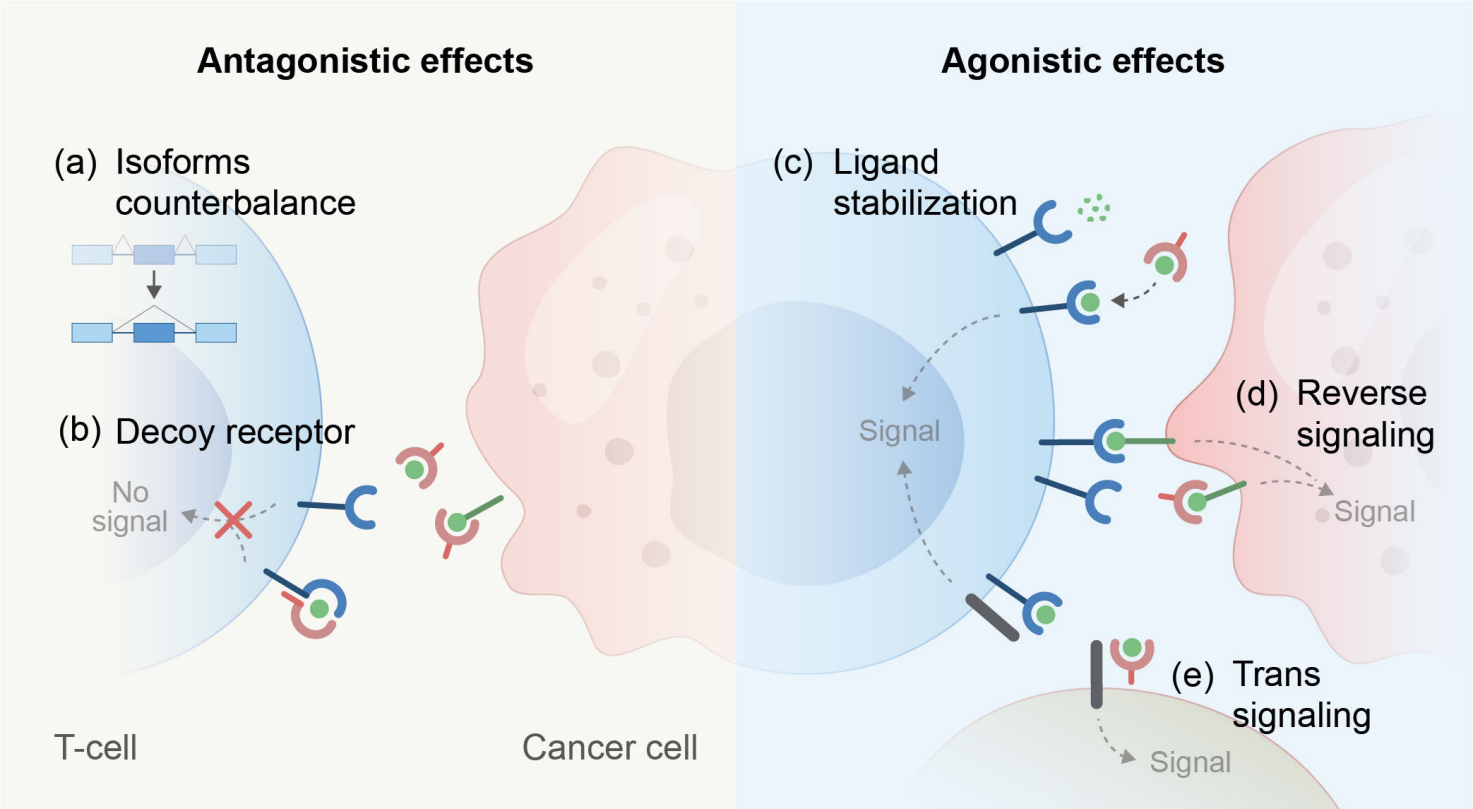
Mechanisms that lead to antagonistic or agonistic effects of soluble ectodomains derived from immune regulatory receptors. (**A–E**, mechanisms depicted).

A prevalent splicing pattern observed in immune receptors gives rise to soluble isoforms that lack membrane anchorage and are secreted into the extracellular space. These soluble receptors may regulate signaling cascades, which differ from those initiated by their parental receptor (**Table 1**). Most prominently, the soluble receptors can function as a decoy of their corresponding ligands and compete with their constitutive, membrane-bound forms (26–35). The ratio between the membranal and the soluble isoforms of a receptor can remain fixed (26, 36). However, it might change depending on the cell’s metabolic, functional, or differentiation state (37, 38). Diverting the pre-mRNA splicing towards the soluble isoforms results in reduced expression of the membrane-bound receptor and can even negate its cellular effect. In a different context, the soluble receptors may have agonistic effects (28, 39) and initiate reverse signaling by binding to other receptors (40–42). For example, glucocorticoid-induced tumor necrosis factor receptor (GITR) ligand that is expressed on plasmacytoid dendritic cells prompts a reverse signal that initiates noncanonical NF-kappaB-dependent induction of indoleamine 2,3-dioxygenase upon binding to soluble GITR. This leads to the tryptophan catabolism immunoregulatory pathway (43). In addition, soluble isoforms have been documented to bind with their ligand to distinct membranal partners, activating trans-signaling pathways (44, 45). Furthermore, some soluble receptors stabilize their ligand configuration or alter their biodistribution (31, 45–47). Another typical example of important alternative splicing of immune cells is the removal of the hydrophobic transmembranal segment of the B-cell receptor to form a secreted immunoglobulin (48). Interestingly, soluble receptors may exert different functions (i.e., agonistic and antagonistic) depending on their concentration (45, 46, 49) (**Figure 2**).

**Table 1:** Soluble T-cell immune receptors due to alternative splicing.

Superfamily	Name	Splicing event	Suggested mechanism	Function	References
**Immunoglobulin superfamily**	BTLA	TMD skipping	Unknown	Increases cellular proliferation	(95, 96)
	CD28	TMD skipping and premature stop codon	Unknown	Inhibits T-cell proliferation induced by anti-CD3 antibodies or by mitogens	(97, 98)
	CD83	TMD skipping	Binds MD2 on monocytes	Inhibits DC-mediated T-cell stimulation, proliferation, and IL-2 secretion	(40, 42, 50, 99)
	CTLA-4	TMD skipping and premature stop codon	Binds B7 on APCs	CTLA-4 agonist, inhibits the immune response	(28, 39, 58)
	ICOS	ICD skipping	Binds ICOSL	Inhibits T-cell proliferation	(29)
	LAG-3	Alternative 5’ splice site and premature stop codon	Unknown	Controversial	(47, 100, 101)
	PD-1	TMD skipping	Binds PD-L1 and/or PDL2	Enhances immune cell response: (a) Block PD-1/PD-L1 interaction. (b) Reverse signaling into DC.	(26, 41, 69)
**Interleukin receptors**	Common γ chain	TMD skipping and premature stop codon	Binds IL-2Rβ and IL-7Rα	Antagonizes IL-2 and IL-7 signaling	(27)
	IL-1RAcP (co-receptor)	Exon skipping and premature stop codon	Binds IL-1RII and increases its affinity for IL-1α and IL-1β	Negative regulation of IL-1 signaling	(32, 38, 102)
	IL-4Rα	Exon inclusion and premature stop codon	Binds IL-4	Both neutralizing and stabilizing IL-4	(31, 57, 103)
	IL-6Rα	TMD skipping	Binds IL-6	(a) Stabilizes IL-6 (b) sIL-6Rα/IL-6 trans-signaling via membranal IL-6ST	(44, 47, 54, 94, 104, 105)
	IL-6ST, gp130 (co-receptor)	Exon skipping and premature stop codon	Bind sIL-6Rα/IL-6	Prevents sIL-6Rα/IL-6 trans-signaling	(106)
	IL-7Rα	TMD skipping	Binds IL-7	(a) Competes with membranal IL7R. (b) Decreases IL-7 early consumption and results in prolonged availability and increased IL-7 bioactivity	(33, 45)
**TNFR superfamily**	TNFR2	TMD skipping and premature stop codon	Binds TNF	High concentrations inhibit TNF signaling. Low concentrations stabilize TNF trimeric form	(46, 49, 59)
	TNFRSF6, FAS	TMD skipping	Competes with mFAS for FASL binding	Prevents cell death	(30, 52, 53)
	TNFRSF9, 4-1BB	TMD skipping, alternative 3’ splice site, and premature stop codon	Competes with mCD137 for CD137L binding	Reduced T-cell proliferation and IL-2 secretion	(34, 35)
	TNFRSF18, GITR	Exon skipping and premature stop codon	Binds GITRL	Reverses signaling via membranal GITRL and proinflammatory effect	(56, 107)
**TGF beta receptors**	TGF-β Type II Receptor	Alternative 3’ and 5’ splice site - premature stop codon	Binds TGF-β	Inhibits the canonical TGF-β signaling pathways	(93, 108)

The common splicing patterns that lead to the generation of soluble isoforms of membranal receptors include (1) *Transmembrane domain (TMD) skipping*: In the process of alternative splicing, skipping of the transmembrane encoding exon results in the creation of a soluble product encompassing both the intracellular and extracellular domains (26, 33, 50–55); (2) *Alternative terminator*: a shortened soluble isoform is encoded by a sequence that includes a mutually exclusive exon containing an alternative polyadenylation site, or by alternative splicing that results in frameshift and premature stop codon (48, 56, 57). As a result, the translated proteins include only the extracellular domain, lose their membrane anchorage and become soluble (27, 58, 59).

**Figure 3:**
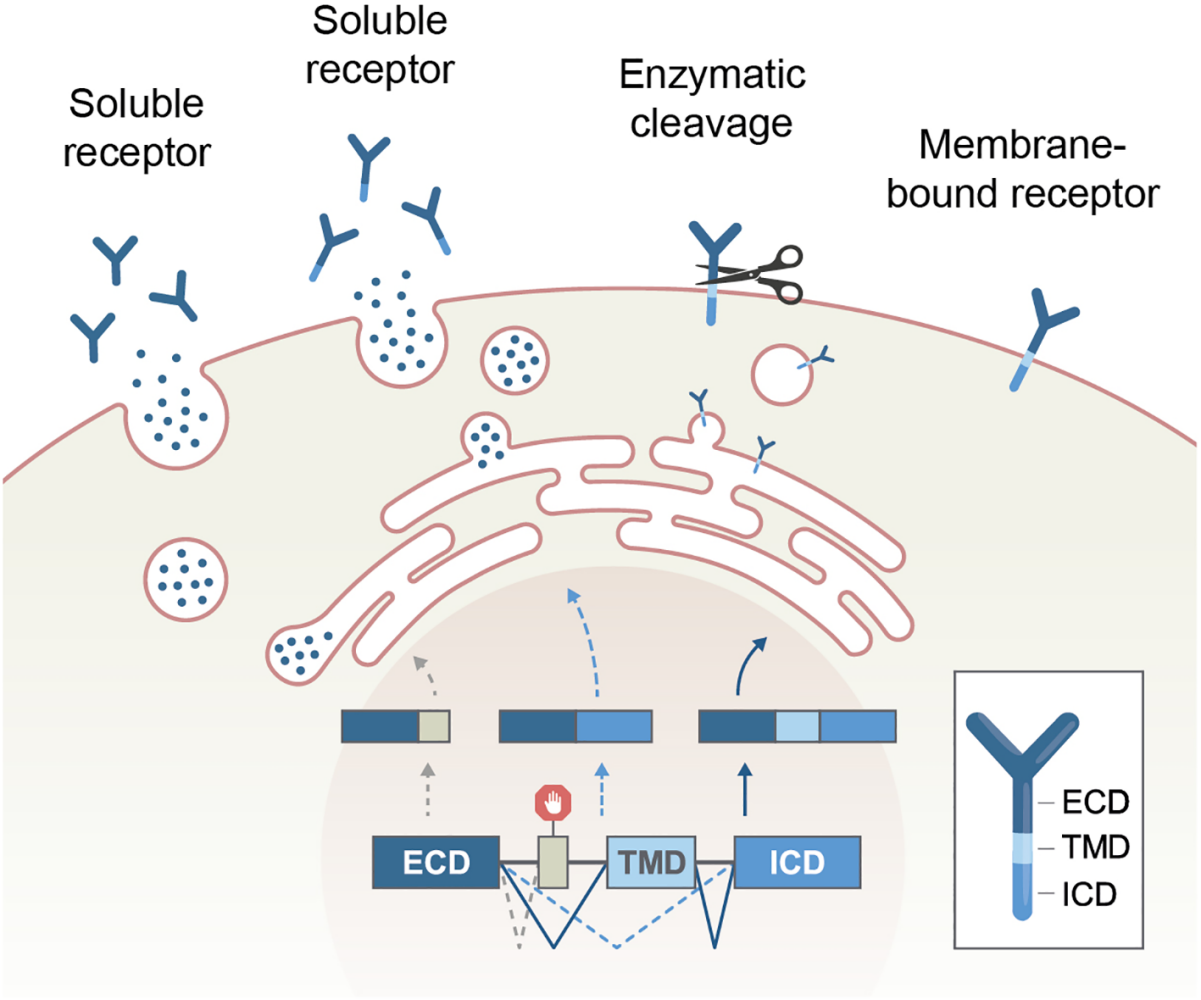
Production of soluble receptors by alternative splicing or enzymatic cleavage. Of note, the intracellular part of a receptor remains only in the cleavage process. ECD, extracellular domain, TMD, transmembrane domain, ICD, intracellular domain.

It should be noted that alternative splicing is not the sole mechanism that creates soluble receptors. These segments can also be made by proteolytic cleavage of extracellular domains by proteases in the extracellular matrix (**Figure 3**). However, unlike AS, the intracellular domain (ICD) of a cleaved receptor remains anchored and theoretically may retain its effect. The impact of a truncated signaling domain is diverse or unknown. Typical examples of receptors that utilize both mechanisms to produce their soluble formats are cytokine receptors, including the TNF and TNFR superfamily (59, 60). In addition, some immune receptor genes lack the transmembrane domain and are, therefore, constitutively expressed as soluble receptors with linked intracellular and extracellular domains. They mainly function as decoy receptors (61). For example, decoy receptor 3 (DcR3, TNFRSF6B) is a secreted TNFR superfamily member that lacks a transmembrane domain. DcR3 can interrupt FAS-FASL interaction by binding FASL and inhibiting FASL-induced apoptosis (62).

### Alternative splicing of the immunoglobulin superfamily

The immunoglobulin (Ig) superfamily is a large group of proteins with a common Ig domain. The Ig superfamily is critical in the immune response networks (63). Some Ig superfamily receptors can be translated to a soluble form by an alternative splicing process (64, 65). Among these are CTLA4 (39, 58), CD83 (42, 66), and LAG3 (66). Here, we will focus on two specific examples: The programmed cell death receptor PD-1 and the B cell receptors (BCRs) that convert, after splicing, into immunoglobulins (antibodies).

#### PD-1

Following stimulation, PD-1 is expressed on T-cells in a membrane-bound form (mPD-1). When it binds to its ligand (PD-L1), mPD-1 inhibits the effector functions of T- cells, promotes apoptosis, and restricts proliferation (67, 68). PD-1 exon 3, which encodes the transmembrane domain, can be skipped by alternative splicing, generating a soluble receptor form (sPD-1). The ratio between the two isoforms is consistent during T-cell activation (36). sPD-1 can act as a decoy receptor and compete with the PD-1 receptor on the interaction with the ligands PD-L1 and PD-L2, and block the interaction of PD-L1 with B7-1 (69). The shedded PD-1 ectodomain exerts a similar effect, suppressing the PD-1 inhibitory function (70, 71). It has been speculated that sPD-1 has a reverse signaling effect when binding to PD-L1 on dendritic cells (41).

**Immunoglobulins** can be membrane-bound or secreted as antibodies. Naive B cells express membrane-bound receptors, usually from the IgM class. Following stimulation, the B cell receptors undergo alternative splicing via an alternative terminator mechanism. As a result, the carboxy terminus no longer contains the hydrophobic transmembrane domain but, instead, has a hydrophilic secretory tail. The secreted antibodies play a crucial independent role during the immune response (48).

### Alternative splicing of the TNF-receptor superfamily

The TNFRSF comprises trimeric receptors made of three homologous molecules that initiate signaling pathways involved in inflammation, proliferation, differentiation, cell migration, and induction of cell death (72). Like the Ig superfamily, TNFRSF members share similar splicing patterns that result in soluble isoforms.

FAS (CD95, TNFRSF6) is one of the best-known members of the TNF receptors superfamily. It is abundantly expressed in many tissues, including the gastrointestinal tract, the respiratory system, and lymphoid tissues (73–75). FAS is mainly known for its pro-apoptotic pathway activation following FAS ligand (FASL) binding (76). However, it also has other functions, e.g., it takes part in the differentiation of naïve T cells to memory cells (77). FAS is robustly expressed on T-cells and has an apoptosis-inducing role during T-cell development (78) and activation (79–81). A specific alternative splicing event is the skipping of exon 6, which encodes the transmembrane domain of FAS, resulting in a soluble form of the FAS receptor (52, 53). The FAS exon 6 skipping mechanism has been studied extensively. It has been shown that many splicing factors can regulate this event, among them TIA-1 (82), PTB (82), HuR (83), hnRNP A1 (84), SRSF4 (85), SRSF7 (86), and SRSF6 (87). Similarly to PD-1, the soluble FAS receptor competes with membrane-bound FAS for FASL ligation, thereby limiting FAS signaling (30, 52). Bajgain et al. described the ability of secreted FAS extracellular domain to enhance CAR T-cell antitumor activity against a FAS-ligand-expressing tumor (88).

TGFβ (transforming growth factor beta receptor) TGFβ is of special interest because it controls immunity via a rich network of cells and mediators, with the end result being immune evasion of the cancer tissue. The biological functions of TGFβ are mostly mediated by the monomeric, soluble form of the protein. The monomer is cleaved by proteases in the Golgi complex and later released from glycoproteins that ligate it in a non-covalent manner (89). TGFβ enhances the expansion of regulatory T cells (Tregs), the inhibition of NK and effector T cells, and the induction of immune suppressive cytokines including IL-4 and IL-10. Active TGFβ exerts its effect via receptors that activate SMAD transcription factors, a family with hundreds of regulatory elements. Tumors exploit TGFβ to induce a supportive stroma that weakens the immune response by acting as a mechanical barrier and expressing inhibitory membranal ligands, such as PD-L1 (90, 91). In a series of patients with gynecological cancers who received immune checkpoint inhibitors, a high TGFβ expression score correlated with treatment failure and reduced survival (92). The type II receptor for TGFβ has a splicing variant which lacks the transmembrane domain, and exert a higher binding affinity to the three sub-types of TGFβ. By doing so, it competes with the natural ligands and reduces fibrotic pathology (93).

### Type I cytokine receptors

In addition to the Ig and TNFR superfamilies, members of other immune receptor families can generate soluble forms through alternative splicing, including type I cytokine receptors IL6Rα (54, 94), IL-4Rα (57), and IL-7Rα (33). Another example is the common γ chain IL-2, 7, and 15 cytokine receptors, for which skipping exon 6 encoding the transmembrane domain results in a frameshift and a premature stop codon. The resultant proteins contain only the extracellular domain. The soluble IL-2 and IL-7 receptors were reported to impair T-cell signaling and function (27).

## Pathology of splicing and alternative splicing

Splicing is an imperative regulator of most cellular functions. Therefore, disrupted splicing regulation can lead to different pathologies, depending on the involved tissue and the protein products of the aberrant transcript. The most investigated pathologies that result from erroneous splicing events include neurodegenerative disorders and cancers. The first arise from germline mutations, while the latter arise from somatic genome aberrations. However, splicing-related mutations can cause many other disorders, such as dilated cardiomyopathy and Marfan syndrome (109, 110).

It remains a mystery why germline splicing-related mutations primarily affect the brain. One theory holds that alternative splicing is crucial in determining the neural cell state (19) and that neural tissue is rich in tissue-specific splicing events. However, not all splicing-related mutations in neural cells lead to a change in alternative splicing. An example of this is Duchenne muscular dystrophy (DMD), where a deletion of an exon leads to the production of a truncated protein via the process of nonsense-mediated decay (NMD) rather than a new isoform of the original protein (111).

Some argue that 15-50% of pathological mutations affect gene splicing (9–11). Nevertheless, these diseases are not regarded as splicing-related disorders since mutations that do not change the coding sequence are typically misclassified as allelic variations (112–114). In addition, the wide use of exome sequencing, which filters out most intronic parts, introduces an inherent bias underscoring splicing mutations (115–118).

### Dis-regulated splicing leading to neurodegenerative disorders

Neurodegenerative diseases are a group of disorders caused by the gradual loss of neuronal cell function or structure. Strikingly, splicing-related mutations are one of the leading causes of many neurodegenerative diseases (119). The most investigated neuronal disorder instigated by splicing is spinal muscular atrophy (SMA). Nonetheless, most neural pathologies, such as early-onset Parkinson’s (119–121), Alzheimer’s disease (122), familial dysautonomia (123), and Amyotrophic Lateral Sclerosis (ALS), could evolve from splicing mutations (124, 125).

Given the significant number of neurodegenerative disorders caused by mutations impacting RNA splicing, it is not surprising that there have been numerous efforts to investigate the use of splicing-editing techniques as a treatment option for these conditions. For example, antisense oligonucleotides (ASOs) are being widely researched for their potential use in treating SMA, DMD, and ALS (126). The use of ASOs to manipulate alternative splicing is further discussed elsewhere in this review. The use of CRISPR-Cas9 to affect alternative splicing has been suggested for Duchenne muscular dystrophy (127), and spliceosome-mediated RNA trans-splicing (SMaRT) (128) has been tested in Huntington’s disease (129), DMD (130), and Alzheimer’s disease (131).

### Dis-regulated splicing causing cancer and immune evasion

**Figure 4:**
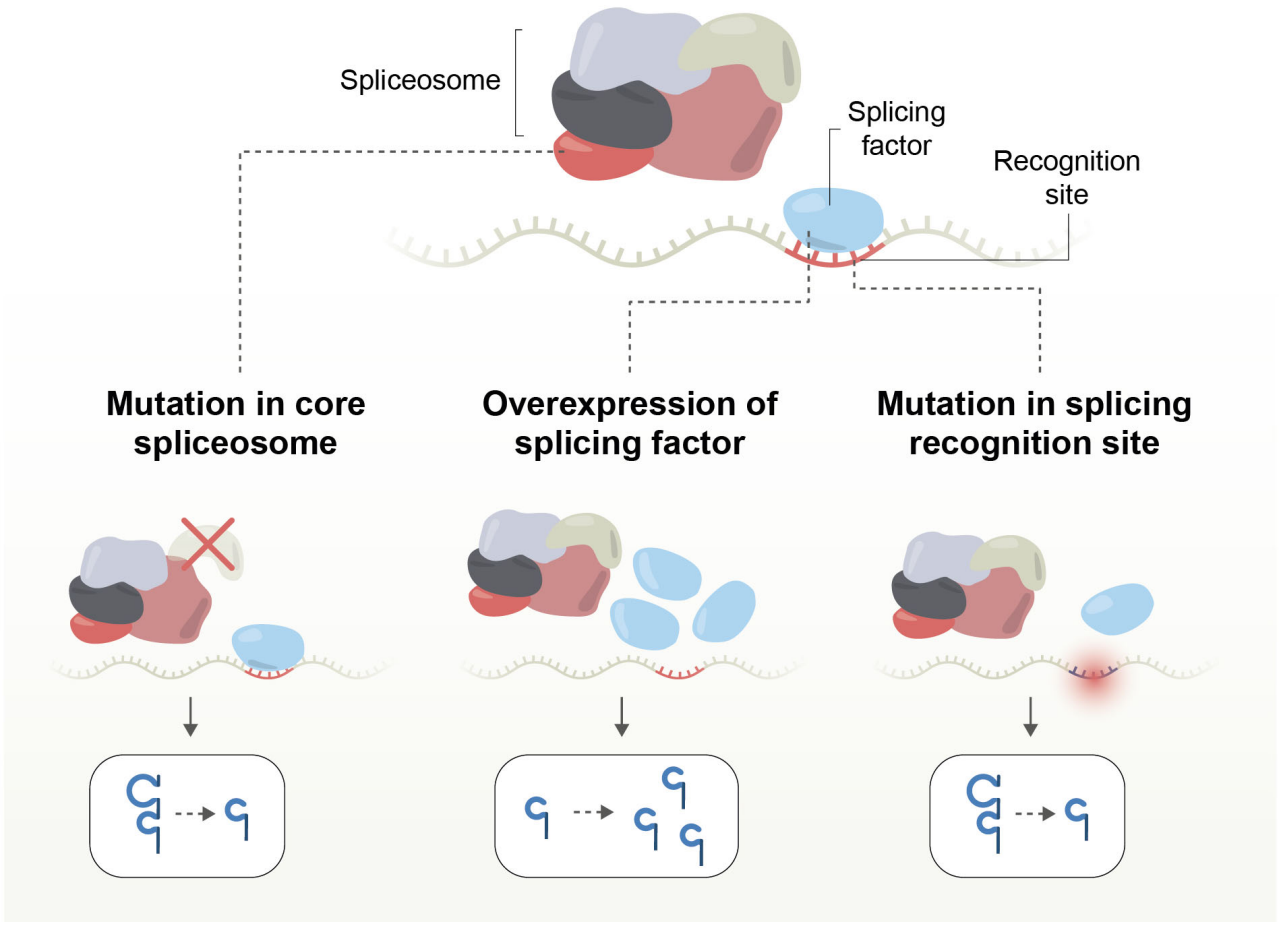
Mechanisms of splicing disruption by mutations affecting the core spliceosome complex; splicing factors, or splicing recognition sites, altering the expression level of a single gene or creating new isoforms.

Splicing-related mutations in cancer can be grouped into three categories: 1-those affecting the core spliceosome complex, resulting in new isoforms; 2-those impacting splicing factors, affecting the expression levels of multiple isoforms; and 3-those affecting splicing recognition sites, altering the expression level of a single gene or creating new isoforms (**Table 2**; **Figure 4**).

**Table 2:** Specific mutations associated with splicing dis-regulation in human cancers.

Mutation type	Mutation	Description	Cancer	References
Mutation in the core of spliceosome complex	U1	Characterized by different binding to the 5’ splicing site	Over 30 types of malignancies, including hepatocellular carcinoma, chronic lymphocytic leukemia, medulloblastoma	(132, 133)
	U2AF1	Leads mostly to exon skipping and 3’ alternative splicing in specific genes.	Hematological, pancreatic cancer, and lung adenocarcinoma	(134–139)
Changes in splicing factors	SF3B1	Alternative branch point selection leads to aberrant/cryptic 3’ splicing sites.	The most common splicing factor mutation in cancer. Common in hematological malignancies, uveal melanoma, breast cancer	(140–147)
	hnRNP A1	Overexpression of hnRNP A1 leads to miss-regulated splicing and increases oncogenic isoforms.	Many types of cancers, including lung, breast, and gastric cancers.	(148–152)
	SRSF1	Overexpression of SRSF1 in tumor cells increases a wide range of genes. Overexpression can be caused by copy number variation or changes in the mRNA level.	Breast, lung, colon, and other tumors.	(153–159)
	SRSF6	SRSF6 is a proto-oncogene that, when overexpressed, leads to an increase in tumor-promoting isoforms.	Skin, colon, lung, and other cancers.	(160–162)
Mutation in splicing recognition sites	MET	Exon 14 skipping mutations in the gene MET leads to a protein missing the phosphorylation site, which impairs protein degradation.	Non-small cell lung cancer (NSCLC)	(163–168)
	MLH1	Some of the mutations associated with HNPCC are missense/nonsense splicing-related mutations.	Colorectal cancer (CRC)	(169–174)
	TP53	2-4% of the mutations in TP53 are mutations in intronic splicing sites, which can lead to a truncated protein or a shift towards oncogenic splice isoforms. In addition, many other mutations in the gene can effect specific isoforms of TP53.	All tumors bearing TP53 mutations	(175–179)

Splicing factors mutations are particularly prevalent in myeloid neoplasms; for example, SF3B1, that increases anti-apoptotic isoforms, enhances tumor proliferation and progression, and is associated with poor survival of patients (134, 140, 141, 180, 181). U2AF1 is another splicing factor mutated in myeloid malignancies that drives altered splicing preferences. Intronic mutations are more frequent than exonic, and a third of somatic mutations in the exon-intron boundary are associated with splicing changes. If a mutation occurs in a 5’ or 3’ splicing site, there is a greater than 50% chance of it leading to a splicing shift (182).

Splicing can be employed as a cancer treatment approach in various forms: using single-stranded oligonucleotides to change the splicing of specific genes and switch between oncogenic and tumor-suppressing forms, as has been demonstrated for the BCL gene (67); regulating specific splicing factors through drugs that directly impact them, such as blocking SF3B1 (68); or by attacking the pathway which the mutant splicing factor exploits. Thus, tumors with driver mutations in SF3B1 or U2AF1 may be vulnerable to NMD inhibition (68–72). Some widely used therapies, such as camptothecin and cisplatin, have been found to impact RNA splicing, potentially contributing to their efficacy (73–75).

As discussed in the following paragraph, recent attention has focused on the generation of neo-antigens by including erroneous transcripts. However, altered splicing and the emergence of usually unexpressed isoforms independently impact tumor immunogenicity. These effects often hinder the anticancer immune response. For example, HLA tumor-enriched alternative splicing events occur in 10-30% of lung and breast cancers, affecting MHC expression. When HLA expression is inconsistent, the ability of tumor epitopes to be presented and recognized is diminished or completely lost (183). In ovarian cancer, certain splicing factors, such as BUD31, SF3B4, and CTNNBL1, may indirectly support immune evasion (184). This immune escape may involve increased PD-L1 expression and primary resistance to PD-1 inhibitors. Such mechanisms are seen in clear renal cell carcinoma, where an exon-including splicing event in the chromatin remodeling gene PBRM1 contributes to immune evasion (185).

### Generation of cancer neo-antigens by mutations in splicing factors

While reports indicate that altered splicing isoforms contribute to tumor immune evasion, splicing alterations are now attracting significant interest as a source of cancer antigenicity. This interest stems from the potential of AS to drive isoforms that include retained intronic sequences. These intronic transcripts, in turn, may form neoantigens—peptide sequences that have not had the opportunity to tolerize the immune system. Such newly transcribed sequences hold the potential for generating protective immunity and improving clinical responses to immune checkpoint inhibitors (61–64).

Several pharmacological compounds have been used that either degrade splicing factors, disrupt spliceosome assembly, or inhibit nonsense-mediated decay (186, 187). One example is indisulam, an anticancer sulfonamide that generates aberrant transcripts. Interestingly, indisulam does not directly inhibit cancer growth; instead, it triggers a T-cell response against cryptic sequences from abnormal RNA, which impedes tumor progression. Other splicing-disruptive compounds, such as pladienolide B and H3B-8800, are currently being evaluated in experimental systems and clinical trials for myeloid neoplasms. Predicting the effect of splice manipulation on the tumor microenvironment is challenging, but as will be discussed, induction of soluble ectodomains from immune-modulatory receptors may interfere with immune checkpoint inhibitors. Soluble PD1, for instance, may saturate PD-1 blocking antibodies and reduce their availability to rescue exhausted antitumor T cells (188).

## RNA sequencing for splicing analysis

The technology developed to sequence RNA and obtain long transcript reads that capture added or missing nucleotides was crucial to assessing AS in health and disease.

**Figure 5:**
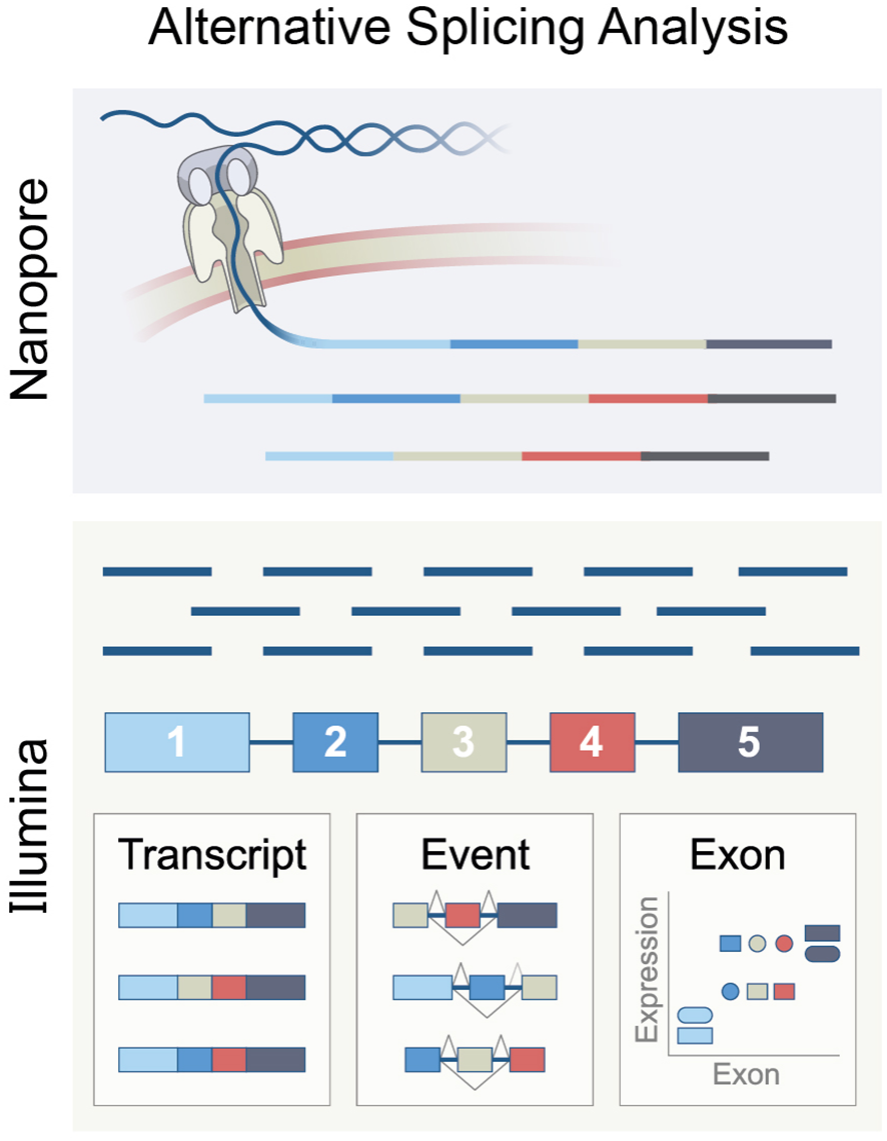
The principle of RNA splicing analysis using Nanopore long-reads or Ilumina short-reads, representing methods based on exon, isoform, or event.

Bulk RNA sequencing (RNA-seq) is mainly performed using two methodologies (**Figure 5**). The first is short-read sequencing, which can sequence RNA molecules in reads of up to 301 base pairs (bp), for which the Illumina platform is commonly used. The second is long-read sequencing, known as “third-generation sequencing.” This method can sequence up to 26,000 bp RNA molecules in the NanoporeTM platform (189). Long-read sequencing has the advantage of identifying full-length transcripts derived from each gene. However, this sequencing method had an accuracy of only 90% and is, therefore, error-prone. Erroneous sequencing interferes with the alignment of the reads to a reference genome and thus can miss sutured exons and their splice junction, an important feature required to determine the splicing pattern (190). However, recently, Nanopore announced that its sequencing accuracy has increased to 99.9%.

Since Illumina sequencing is well-established and widely used, most splicing analysis tools are designed for short reads. Analyzing bulk RNA-seq from Illumina data can be done in three ways. The first is determining the exon expression level and comparing its expression in varying biological settings or states. This method is called “exon-based”. The second method aims to deduce isoform expression from the short reads sequencing. This method is called “isoform-based”. The third approach, called “event-based,” computes the relative inclusion of an exon between two exons. This approach utilizes reads of splice junctions that overlap at least two exons.

A comparison of the main computational tools based on these three methods concluded that the event-based and exon-based tools while having a relatively low overlap, seem to work the best. It is suggested that concurrent use of the two methods yields the optimal splicing map of a given cellular population (191).

Another critical parameter to consider when performing splicing analysis is the quality of the RNA-seq data. In this regard, two features need to be accounted for: the depth of the sequencing and the length of the reads. Mehmood et al. (191) have noted that a depth between 40-60 million reads per sample will be sufficient for a robust splicing analysis. When considering reading length, 100 bp reads were the threshold for thoroughly detecting splicing junctions (192). It is also advised to sequence the data using paired-end sequencing to increase the read length.

Extracting splicing data from single-cell RNA-seq is even more complex. In general, to apply splicing analysis tools, the samples must be produced to capture the full transcript. However, most single-cell RNA-seq technologies are based on a 3’ or 5’ capturing of the RNA molecule. As a result, while preparing the sequencing libraries, only the transcript’s end is included; thus, there are limited options for splicing analysis (193). The main exception to these technologies is Smart-seq sequencing, which captures reads from all over the transcript. This technology enables splicing analysis with the limitation of read depth and length. This was demonstrated with the single-cell splicing analysis tool ‘Expedition.’ In their study, Yan Song et al. (194) used Smart-seq2 sequencing with a mean of 25 million reads per cell and a 100 bp read length. In comparison, 10XGenomics™ recommends a sequencing depth of 20,000-50,000 reads per cell and a read length of 28 bp (195); this is shallow sequencing compared to Yan Song’s analysis. To overcome these problems, a new single-cell long-read RNA sequencing technology based on Pacific Bioscience’s sequencing, called MAS-ISO-seq, was recently introduced. This technology is still new and needs further investigation. Furthermore, a joint project of Nanopore and 10x Genomics produced long-reads in single-cell RNA sequencing (196).

## Splicing modification using antisense oligonucleotides

During the 70s, evidence accumulated for the promising ability of small RNA molecules to control translation processes (197–199). Paterson et al. were the first to generate a translation-inhibiting system based on a complementary mRNA-DNA hybrid, resulting in reversibly arrested β globin translation (199). In 1978, Paul Zamecnik and Mary Stephenson used synthetic DNA against RSV (200). Their 13-nucleotide product hybridized with the viral mRNA and prevented viral replication (201). Later, it was shown that the mechanism of action of the short, single-stranded oligonucleotides included RNase-H1 assembly to the DNA-mRNA hybrid, cleavage, and mRNA degradation (202). Despite the promising results, the research in this field was paused for a decade, mainly due to technical issues related to nucleotide synthesis (203, 204), skepticism about the ability of nucleic acids to enter target cells, and restricted knowledge of the human genome (205). The progress in these aspects re-ignited the research in nucleic acids-based manipulation. The chemically modified, short, single-stranded antisense oligonucleotides (ASOs) improved durability, cellular uptake, delivery, and post-transcriptional effects.

### Chemical modifications

**Figure 6:**
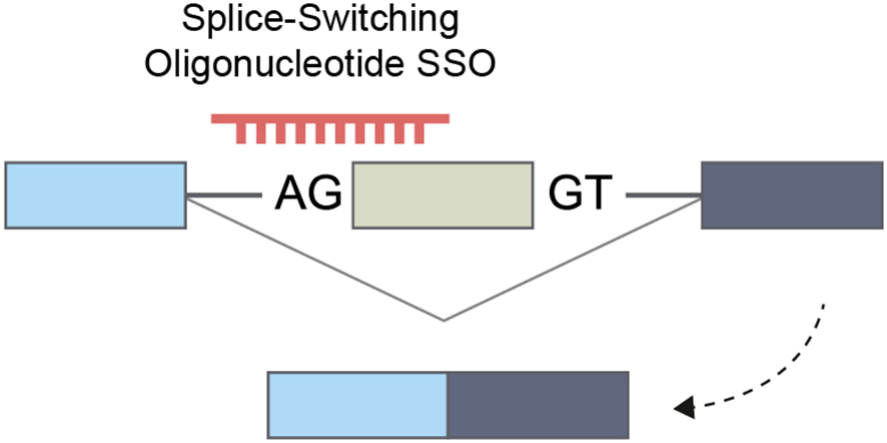
Splice-switching oligonucleotide that enhances exon skipping and increases the expression of an alternative isoform.

The advancement of chemical modifications of nucleic acids marked a significant milestone in the clinical application of this compound class. A key outcome was the development of splice-switching antisense oligonucleotides (SSOs), designed to modify alternative splicing patterns and enhance exon skipping. Specifically, chemical modifications that reduce RNaseH activity form a stable DNA-mRNA hybrid, preventing subsequent RNA degradation (206). These SSOs can be directed towards splice-site sequences, hindering and redirecting the spliceosome to an alternative splice site in the subsequent exon (207, 208) (**Figure 6**).

In addition to splicing alterations via complementation to splice sites, SSO can modify splicing by targeting splicing enhancers (ESE, ISE) or silencers (ESS, ISS) (209, 210). These interventions may interrupt splicing by inhibiting linkage to splicing factors, leading to exon exclusion or inclusion. In addition to whole exon skipping, the pre-mRNA splicing modulation can result in intron retention, alternative 5′ and 3′ splice sites, alternative promoter, or alternative polyadenylation sites (209).

Finding SSO-targetable splicing motifs is not trivial. A systematic scan of the exon of interest is necessary to spot the precise sequence, which the SSO should complement to alter the wild-type splicing pattern.

**Figure 7:**
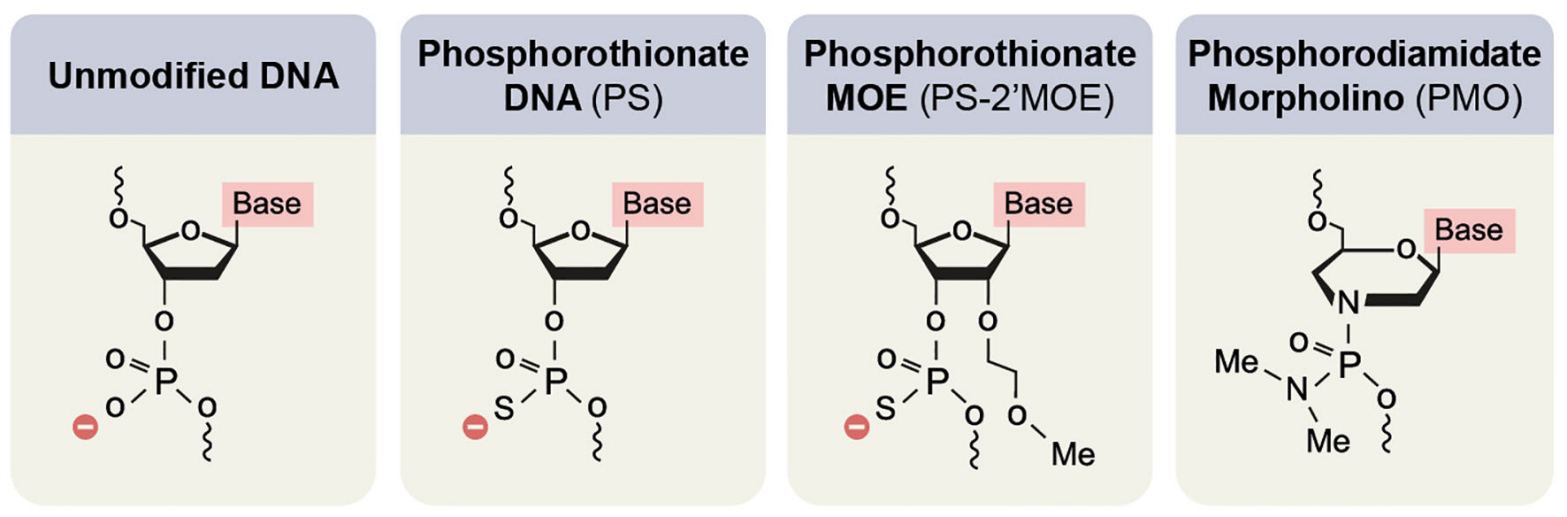
Main chemical modifications of nucleic acids to improve the clinical applicability of oligonucleotides.

Two types of chemical modification are currently used for FDA-approved drugs: 2′-O-methoxyethyl (2′-MOE) nucleosides with phosphorothioate (PS) backbone and phosphorodiamidate morpholino oligomers (PMO) with a N, N-dimethylamino phosphorodiamidate backbone (**Figure 7**).

2’-MOE belongs to a group of modifications in the 2’O of the furanose ring of the nucleic acid. Other prevalent modifications are 2′-O-methyl (2′-OMe), locked nucleic acid (LNA), and SSOs containing 2′-constrained ethyl (2′-cEt). Alongside the RNaseH1 resistance, the 2’O modifications increase the SSO affinity (211). High affinity is attributed to higher potency, longer half-life, and less immune-provoking properties (212, 213). 2’MOE modifications are usually accompanied by switching Oxygen in the backbone to Sulfur (PS). This switch decreases the SSO affinity but improves the resistance to nuclease activity (214, 215) and molecular binding to proteins – resulting in reduced kidney clearing (216) and improved uptake by target cells (217–219).

In PMO (220), a morpholino ring replaces the furanose ring. In addition, the negatively charged backbone is replaced by a N, N-dimethylamino phosphorodiamidate backbone. As a result of these changes, the SSOs have higher *in vivo* tolerance but faster kidney clearance, which requires a higher dosage (221, 222).

Although these are the main modifications currently used for SSO drugs, recent publications have shown how additional chemical modifications can further improve splicing modulation. For example, Langner et al. synthesized a hybrid that combines PMO modification with a PS backbone, which exhibits higher efficiency than 2’-MOE modification with the same backbone (223).

#### SSO-based drugs and clinical trials

Eighteen RNA-targeted oligonucleotide drugs have been approved, including five SSOs (206). The most advanced examples of clinical use of SSOs are in the field of genetic neuromuscular diseases.

The first SSO that the FDA approved is used for spinal muscular atrophy (SMA) treatment. The drug nusinersen, approved by the FDA in 2016, is an SSO with a 2′-MOE modification and a PS backbone. Nusinersen targets the splicing silencer located in *SMN2* intron 7 pre-mRNA, and by blocking the binding of hnRNPA1 and A2, it promotes higher exon 7 inclusion, increasing the SMN2 protein synthesis (224, 225). The treatment results in prolonged survival and a dramatic improvement in motor development.

Other approved SSO drugs are used for the treatment of Duchenne muscular dystrophy (DMD). This severe, progressive muscle-wasting disease causes difficulty in movement and breathing and, eventually, early death. It is caused by mutations in the DMD gene, leading to impaired dystrophin protein production (226). In recent years, the FDA has approved four drugs based on a mechanism of SSO with PMO modification. The first drug approved, eteplirsen, was approved in 2016 and causes mutated exon 51 skipping (227). Three additional drugs that work in a similar mechanism have been approved in recent years for different mutations that lead to DMD: golodirsen, which causes exon 53 skipping, was approved in 2019 (228); viltolarsen, approved in 2020, also causes exon 53 skipping (229); and casimersen, approved in 2021, induces exon 45 skipping (230). To date, DMD is the only disease for which even modest, consistent clinical benefit has been shown using PMOs. Thus, PMO SSOs have demonstrated minimal and doubtful applicability in mammalian systems (227, 231).

Considering the achievements of SSOs in DMD and SMA, several groups have recently published promising data demonstrating the potential of ASO in other diseases. For example, Yang et al. (232) show the use of SSO to prevent a splicing pattern that arises from an alternative 3’ splice site between SYNGAP1 exon 10 and exon 11. This splicing pattern leads to nonsense-mediated decay (NMD). Mutations in this gene are a common cause of autism and intellectual disability. Using SSO with 2′-MOE modification increased the expression of the active protein in an *in vitro* system. Promising results for the use of SSO can also be seen in the treatment of Dravet syndrome (233), Huntington’s disease (234), and fragile X syndrome (235). Similarly, in cystic fibrosis, Oren et al. (236) and Michaels et al. (237) demonstrate the use of SSO that leads to mutated exon 23 skipping, increasing the expression of the CFTR protein.

#### SSO and cancer treatment

The use of SSO in cancer treatment is still in its early stages. There is currently no approved drug, but there are ongoing research studies. The primary approach for anticancer SSO is modulating the alternative splicing of oncogenes toward NMD, nonfunctional dominant negative isoforms, or isoforms with the opposite function.

For example, Dewaele et al. (238) used PMO-modified SSO for MDM4 exon 6 skipping, resulting in nonsense-mediated decay and rescue of MDM4’s target - the tumor suppressor protein p53. The SSO administration reduces diffuse large B cell lymphoma growth both *in vitro* and *in vivo*.

Using SSOs for translatable alternative splicing isoforms was shown in the human epidermal growth factor receptor 2 (HER2) case. HER2 is an oncogene and established therapeutic target in a large subset of women with breast cancer (239). Wan et al. (240) and Pankratova et al. (241) used SSOs to skip HER2 exons 15 and 19, respectively. The manipulations resulted in the upregulation of Δ15HER2, a HER2 inhibitor isoform, and Δ19HER2, a dominant negative isoform, leading to apoptosis and inhibition of proliferation.

Khurshid et al. (242) recently proposed using SSO for patients with rhabdomyosarcoma (RMS). In their article, the group describes the modification of the insulin receptor splicing pattern by targeting the binding site of the splicing factor CELF1. This prevents the skipping of exon 11, leading to an increase in the expression of the receptor in its full form (IR-B). The use of SSO in an RMS cell line system led to a decrease in proliferation, migration, and angiogenesis.

Manipulating cancer-associated metabolic programs using SSO was demonstrated by Wang et al. (243). The group found that elements in exon 10 of the pyruvate kinase M (PKM) gene influence the choice between the inclusion of exon 10 and exon 9. Exon 10 inclusion, the M2 isoform, is common in cancer tumors and is associated with their ability to switch to aerobic glycolysis (Warburg effect). The group demonstrated the possibility of using SSO for splicing modulation in favor of exon 9 inclusion and showed that the manipulation could lead to apoptosis of glioblastoma cell lines. Recently, the group showed a similar effect in a hepatocellular carcinoma mouse system (244). In summary, the use of SSOs to manipulate the immune system is still in its early stages. While there is significant progress in understanding the immune system at the molecular landscape, many complexities regarding the manipulation of T cells are yet to be unraveled.
